# Neuronal tetraploidy in Alzheimer and aging

**DOI:** 10.18632/aging.101312

**Published:** 2017-10-25

**Authors:** José M. Frade, Noelia López-Sánchez

**Affiliations:** Department of Molecular, Cellular and Developmental Neurobiology, Cajal Institute, CSIC, Madrid, E-28002, Spain

**Keywords:** somatic polyploidy, DNA content, entorhinal cortex, cell cycle, E2F1, APPSwe/PS1dE9

Somatic polyploidy can be defined as the increase of the full chromosome complement in specific cells or tissues of a given diploid organism. Although somatic polyploidy is frequently found in invertebrates, evidence indicates that several organs of the human body can also contain somatic polyploid cells, including the liver, heart, blood, and placenta. The vertebrate nervous system is not an exception to this rule, and different subpopulations of projection neurons are known to become tetraploid as they migrate to their final position in the adult brain [1].

Neurodegeneration is often preceded by cell cycle reentry in the affected neurons, which can become tetraploid if they fully replicate their DNA. This may be the case in Alzheimer's disease, a condition in which *de novo* generation of somatic tetraploid neurons has been under debate for years [2]. In this context, we have recently confirmed that the proportion of somatic tetraploid neurons becomes increased in the cerebral cortex of Alzheimer patients, anticipating the normal course of the disease [3]. This increase can be detected in both frontal and parietal cortex already at Braak III-IV, stages of the disease characterized by the absence of neurofibrillary tangles in these structures. Therefore, tetraploidy seems to precede the neuropathological signs of the disease, a view consistent with the observation that forced cell cycle reentry in neurons can lead to neurofibrillary tangle formation and β-amyloid peptide deposition in the mouse brain [4]. This pathological tetraploidization seems to be deleterious as neurons showing increased ploidy at early stages of AD specifically die at later stages [5]. AD-associated neuronal tetraploidization in the frontal and parietal cortex seems to affect differentially to specific neuronal populations, as only one per cent of total neurons (i.e. NeuN-positive cells) undergo *de novo* tetraploidization, while *de novo* tetraploidization is raised by four-five per cent when the MEF2C-positive neuronal population is evaluated.

Somatic tetraploidization of human neurons can also take place during normal aging. In this case it specifically affects the entorhinal cortex [3], a structure involved in memory processing. This suggests that age-associated tetraploidization of neurons could be part of the mechanism leading to cognitive decline with age in humans. Age-associated tetraploidization of entorhinal neurons could also participate in the etiology of AD since neurofibrillary tangle formation is firstly observed in this brain structure before it spreads to other areas of the brain. If this is the case, deciphering the mechanism by which neuronal tetraploidization spreads to the cerebral cortex will contribute to our understanding of this pathology.

Age-associated neuronal tetraploidization can be observed in the murine cerebral cortex as well, taking place in neurons from both superficial and deep cortical layers. Interestingly, this process can be blocked in mice lacking E2F1, a known transcription factor that regulates G1/S transition in proliferating cells. This indicates that the E2F family members actively participate in neuronal tetraploidization, like in differentiating neurons [6]. E2f1^−/−^ mice show improved memory acquisition and consolidation under stringent training conditions, as evaluated by novel object recognition and Morris water maze cognitive tests, thus indicating that neuronal tetraploidization is likely deleterious. In contrast to age-associated neuronal tetraploidization, which affects both superficial and deep layers of the cerebral cortex, neuronal tetraploidization in APP^Swe^/PS1^dE9^ mice seems to preferentially occur in the superficial layers of this structure [3]. It will therefore important to determine whether this observation obtained in a known murine model of AD can be extended to the Alzheimer brain, in which degeneration of neurons located at the superficial layers of the entorhinal cortex are among the first signs of the disease.

Many other questions remain currently unanswered. Possibly the most important one is related to the pathophysiological effects of neuronal tetraploidy. Neuronal tetraploidy is associated with increased cell size [7]. Hence, it is expected that the enlargement of tetraploid neurons could result in physiological changes in these cells, including possible metabolic alterations and critical dependence on oxygen and nutrients. For instance, increased neuronal size is likely dependent of lipids, required for membrane biosynthesis. One could speculate that the ApoE4ε allele, known to codify for a specific version of a Cholesterol protein carrier, could confer increased risk to develop AD due to a hypothetical altered capacity to transport this lipid to *de novo* generated tetraploid neurons. Neuronal tetraploidization could also alter synaptic transmission due to a hypothetical increase of dendritic and axonal size. This could affect the neuronal networks in which these neurons are integrated.

Another important question to be answered is why entorhinal neurons are prone to undergo tetraploidiza-tion in an age-dependent manner. This would be relevant for the design of novel therapeutic approaches against age-associated cognitive loss and maybe also to AD. To respond to this question it would be necessary to understand the molecular basis for neuronal tetraploidy in ageing and neurodegenerative diseases. In this regard, E2F family members arise as promising targets for pharmacological intervention.
